# Discovery of an Antibiotic-Related Small Protein of Biocontrol Strain *Pseudomonas* sp. Os17 by a Genome-Mining Strategy

**DOI:** 10.3389/fmicb.2020.605705

**Published:** 2020-11-26

**Authors:** Kasumi Takeuchi, Wataru Tsuchiya, Zui Fujimoto, Kosumi Yamada, Nobutaka Someya, Toshimasa Yamazaki

**Affiliations:** ^1^Division of Plant and Microbial Sciences, Institute of Agrobiological Sciences, National Agriculture and Food Research Organization, Tsukuba, Japan; ^2^Structural Biology Team, Advanced Analysis Center, National Agriculture and Food Research Organization, Tsukuba, Japan; ^3^Graduate School of Life and Environmental Sciences, University of Tsukuba, Tsukuba, Japan; ^4^Division of Vegetable Production System, Institute of Vegetable and Floriculture Science, National Agriculture and Food Research Organization, Tsukuba, Japan

**Keywords:** comparative genomics, root-colonizing pseudomonads, bacteriocin-like gene cluster, post-translational modification, Gac/Rsm signal transduction pathway, X-ray structural analysis

## Abstract

Many root-colonizing *Pseudomonas* spp. exhibiting biocontrol activities produce a wide range of secondary metabolites that exert antibiotic effects against other microbes, nematodes, and insects in the rhizosphere. The expression of these secondary metabolites depends on the Gac/Rsm signal transduction pathway. Based on the findings of a previous genomic study on newly isolated biocontrol pseudomonad strains, we herein investigated the novel gene cluster OS3, which consists of four genes (*Os1348–Os1351*) that are located upstream of putative efflux transporter genes (*Os1352–Os1355*). *Os1348* was predicted to encode an 85-aa small precursor protein, the expression of which was under the control of GacA, and an X-ray structural analysis suggested that the Os1348 protein formed a dimer. The mutational loss of the *Os1348* gene decreased the antibiotic activity of *Pseudomonas* sp. Os17 without changing its growth rate. The *Os1349–1351* genes were predicted to be involved in post-translational modifications. Intracellular levels of the *Os1348* protein in the deficient mutant of each gene differed from that in wild-type cells. These results suggest that Os1348 is involved in antibiotic activity and that the structure or expression of this protein is under the control of downstream gene products.

## Introduction

Root-colonizing fluorescent pseudomonads are known to be plant-beneficial strains that suppress the growth of other microbes, including plant pathogens, nematodes, and insects. Strains in this group have the potential to produce various secondary metabolites and enzymes with antibiotic activities, which enable them to compete with other microbes, thereby facilitating niche adaptation in the rhizosphere. *Pseudomonas protegens* strains CHA0 and Pf-5 have been used as model strains in research on the biosynthesis and regulation of secondary metabolites by these pseudomonads. They produce 2,4-diacetylphloroglucinol (DAPG), pyrrolnitrin (Prn), pyoluteorin (Plt), hydrogen cyanide (HCN), and exoenzymes (e.g., AprA protease) as their typical exoproducts (Haas and Keel, 2003; Loper et al., 2012). Moreover, since the complete genomic sequence of strain Pf-5 was elucidated (Paulsen et al., 2005), it has provided novel insights into the molecular mechanisms underlying the pseudomonad-mediated suppression of plant diseases. Genomic-guided approaches have facilitated the identification of novel natural products, such as the cyclic lipopeptide orfamide A (Gross et al., 2007), rhizoxin analogs (Loper et al., 2008), and the insect toxin FitD (Péchy-Tarr et al., 2008). Although the suppressive activities of these metabolites against plant pathogenic fungi and oomycetes have been extensively examined, limited information is currently available on their antibacterial exoproducts.

This wide range of secondary metabolites is considered to be a rich source of valuable compounds for industrial applications. We recently screened 48 DAPG-producing strains among 2,800 fluorescent pseudomonads isolated from fields in Japan to identify novel factors involved in the antibiotic activity of newly isolated strain(s). Of these, we fully sequenced three strains, Cab57, Os17, and St29. Cab57 was identified as *P. protegens* based on a 16S rRNA gene analysis (with 100% identity) and whole-genome analysis (Takeuchi et al., 2014), whereas the two other strains were the closest to, but different from *P. protegens* (Takeuchi et al., 2015). We showed that Os17 and St29 exhibited different biocontrol potentials against *Pythium* damping-off and root rot in the cucumber; Os17 was as effective as Cab57, whereas St29 was less effective. A whole-genome comparison of these related strains revealed that Os17 and St29 are the same species and have several strain-specific genomic regions. Among these regions, the complete rhizoxin analog biosynthesis gene cluster (*ca.* 79 kb) was found to be specific to the Os17 genome (Takeuchi et al., 2015). In the *rzxB* (for polyketide synthase, essential for the production of rhizoxin analogs) mutant, growth inhibitory activity against fungi and oomycetes as well as plant protection efficacy were weaker than those of wild-type Os17. Therefore, although we successfully identified anti-oomycete metabolites, it currently remains unclear whether these strains possess antibacterial exoproducts. DAPG-producing pseudomonads are regarded as a source of bacteriocin, as demonstrated in a previous study on strain Pf-5 in which the novel lectin-like bacteriocin LlpA was discovered (Parret et al., 2005).

We also demonstrated that the function of the well-known Gac/Rsm signal transduction pathway (Lapouge et al., 2008; Kidarsa et al., 2013; Valentini and Filloux, 2016) was conserved in Os17, in which the production of rhizoxin analogs was under the control of GacA (Takeuchi et al., 2015). In *Pseudomonas* spp., the GacS sensor kinase is phosphorylated and initiates this pathway, which enables the activation of the cognate GacA response regulator by phosphotransfer, leading to the transcription of non-coding small RNAs (sRNAs) (Kay et al., 2005, 2006; Lalaouna et al., 2012). GacA positively controls the expression of three sRNAs (RsmX, RsmY, and RsmZ) that have high affinity to the RNA-binding repressor proteins RsmA and RsmE, which induces the expression of mRNAs encoding the biosynthetic gene cluster of secondary metabolites (Heeb et al., 2002; Valverde et al., 2003; Kay et al., 2005; Reimmann et al., 2005; Song et al., 2015). The RsmA and RsmE proteins repress the translation of genes by sequestering a conserved **A**N**GGA**N motif in or near to the Shine–Dalgarno sequence and this repression typically occurs during rapid growth (Reimmann et al., 2005; Lapouge et al., 2008). When RsmX/Y/Z sRNAs are induced during restricted growth at a high cell population density, they relieve the translational repression of target genes by sequestering the RsmA and RsmE proteins. Therefore, the biofilm state and secondary metabolism are favored (Lapouge et al., 2008; Valentini and Filloux, 2016). In strain Os17, the *gacA* mutant exhibited the down-regulated expression of *rzxB* and *rzxI*, both of which have the consensus sequence **A**N**GGA**N in their upstream region (Takeuchi et al., 2015). In addition to the wide range of genomic repertoires available for the synthesis of the secondary metabolites described above, fine-tuning of the Gac/Rsm regulatory system is considered to contribute to the versatility of *P. protegens* and related strains for various environmental niches. Previous studies demonstrated that the ability of mutants defective in the Gac/Rsm system to synthesize biocontrol factors, suppress plant diseases, and produce insecticides was reduced (Haas and Défago, 2005; Kupferschmied et al., 2013).

Comparative genomics among closely related strains revealed phenotype-specific genes, thereby enabling us to identify strain-specific biocontrol factor(s). In addition to these strain-specific gene clusters, clusters commonly shared by the genomes of Os17 and St29, but absent from the genomes of *P. protegens*, are also of interest because they may contribute to establishing the uniqueness of this putative novel species. Comparative genomics among the three strains revealed that in addition to 5,321 core genes, 519 coding sequences (CDSs) were specifically shared by the genomes of Os17 and St29, and a number of these CDSs (i.e., 281 out of 519 CDSs) formed gene clusters (Takeuchi et al., 2015). Some clusters were predicted to be involved in the production of secondary metabolites, the function of which remains unknown. In the present study, we focused on one of these gene clusters, Cluster OS3 (consisting of four successive genes *Os1348–1351* in the genome of Os17), which was predicted to be a bacteriocin gene cluster, and we constructed a deficient mutant of each gene. We found that the deficient mutant of *Os1348*, which encodes an 85-aa small protein, exhibited weaker antibiotic activity than wild-type Os17. We also demonstrated that the downstream genes of *Os1348* affected the protein status of Os1348.

## Materials and Methods

### Bacterial Strains and Growth Conditions

The bacterial strains and plasmids used in the present study are listed in Supplementary Table 1. *Pseudomonas* sp. Os17 has been deposited in the NARO Genebank as MAFF212089 (Takeuchi et al., 2015). *Escherichia coli* and *Pseudomonas* strains were routinely grown in NYB [2.5% (w/v) nutrient broth, 0.5% (w/v) yeast extract] and Luria-Bertani (LB) medium with shaking, or on nutrient agar plates [4% (w/v) blood agar base, 0.5% (w/v) yeast extract] amended with the following antibiotics when required: ampicillin, 100 μg/ml; kanamycin, 25 μg/ml; or tetracycline, 50 μg/ml (100 μg/ml for selection of *Pseudomonas* sp.). Incubation temperatures were 28°C for *Pseudomonas* sp. and 37°C for *E. coli.*

In antibiotic assays, bacteria were grown in modified glycerol-casamino acid medium (GCM; Maurhofer et al., 1998) without the amendment of minerals.

### DNA Manipulation

Small-scale plasmid extraction was performed with a QIAprep spin miniprep kit (QIAGEN); large-scale preparations were obtained with a Qiagen plasmid midi kit. Chromosomal DNA from *Pseudomonas* sp. Os17 was prepared using Qiagen genomic tips. DNA fragments were purified from agarose gels with a QIAquick gel extraction kit (QIAGEN). The oligonucleotides used are listed in Supplementary Table 2.

### Generation of Mutants of *Os1348-Related* Genes and the *phlD* Gene

The primers used are listed in Supplementary Table 2. An in-frame deletion in chromosomal *Os1348-related* genes and the *phlD* gene of *Pseudomonas* sp. Os17 was created as follows. Fragments of *ca.* 750–800-bp regions flanking 1348, 1349, 1350, 1351, 1353 and the *phlD* gene were amplified by PCR with the primer pairs 1348UF/1348UR and 1348DF/1348DR for *1348*, 1349UF/1349UR and 1349DF/1349DR for *1349*, 1350UF/1350UR and 1350DF/1350DR for *1350*, 1351UF/1351UR and 1351DF/1351DR for *1351*, 1353UF/1353UR and 1353DF/1353DR for *1353*, or PhlDUF/PhlDUR and PhlDDF/PhlDDR for *phlD*. High-fidelity DNA polymerase KOD Plus^TM^ (Toyobo) and the genomic DNA of *Pseudomonas* sp. Os17 as a template were used for amplification.

Each of the two corresponding fragments were annealed and amplified as a 1.5- or 1.6-kb fragment using the primer pair 1348UF/1348DR, 1349UF/1349DR, 1350UF/1350DR, 1351UF/1351DR, 1353UF/1353DR, or PhlDUF/PhlDDR. These 1.5- and 1.6-kb fragments were cloned into pCR-Blunt II-TOPO^TM^ (Thermo Fisher Scientific). The inserts obtained were confirmed by sequencing. After sequencing, these 1.5- and 1.6-kb fragments were cloned into pME3087 cut with *Bam*HI and *Hin*dIII to give pME3087-1348, pME3087-1350, pME3087-1351, pME3087-1353, and pME3087-phlD, and pME3087 cut with *Kpn*I and *Hin*dIII to give pME3087-1349. These plasmids were mobilized from *E. coli* DH5α to *Pseudomonas sp.* Os17 by triparental mating with *E. coli* HB101/pME497. Excision of the vector via second crossing-over was obtained after enrichment for tetracycline-sensitive cells, generating the *1348*, *1349*, *1350*, *1351*, *1353*, or *phlD* mutant. To delete the *phlD* gene in the *1348-* or *1353-*mutant, the pME3087-phlD plasmid was mobilized to the *1348-* or *1353-*mutant as the recipient strain.

### Complementation of the *1348*-Negative Mutant

To restore *1348* function in the *1348* mutant, a 1.8-kb fragment carrying *1348* was amplified with the primers 3087-1348U and 3087-1348D, high-fidelity DNA polymerase KOD Plus^TM^ (Toyobo), and the genomic DNA of *Pseudomonas* sp. Os17 as a template. The amplified fragment was inserted into pME3087 cut with *Hin*dIII in accordance with the NEBuilder^*R*^ HiFi DNA Assembly protocol (New England Biolabs). The insert obtained was confirmed by sequencing. The resultant plasmid, pME3087c1348, was mobilized from *E. coli* DH5α to the *1348* mutant by triparental mating with *E. coli* HB101/pME497. Excision of the vector via second crossing-over was obtained after enrichment for tetracycline-sensitive cells, generating the *1348* complement mutant. Complementation of the *1348* gene into the chromosome was confirmed by PCR with the primers 1349UF and 1349DR (Supplementary Table 2).

The *1348* mutant was also complemented with the plasmid pME6031-OS3, which contained a 4.4-kb fragment carrying the *1348-1351* genes (Cluster OS3). This fragment was amplified with the primers OS3F and OS3R, high-fidelity DNA polymerase KOD Plus^TM^ (Toyobo), and the genomic DNA of *Pseudomonas* sp. Os17 as a template. The 4.4-kb fragment was cloned into pCR-Blunt II-TOPO^TM^ (Thermo Fisher Scientific). The insert obtained was confirmed by sequencing. After sequencing, the fragment was cloned into pME6031 cut with *Hin*dIII and *Kpn*I. The resultant plasmid, pME6031-OS3, was introduced into the *1348* mutant by electroporation.

### Expression and Protein Purification of the Recombinant Os1348 of *Pseudomonas* sp. Os17 by *E. coli*

The fragment carrying *1348* was amplified from the genomic DNA of *Pseudomonas* sp. Os17 by PCR with the primers 25F1348 and 25R1348 (Supplementary Table S2). The PCR product was inserted into the *Nde*I/*Bam*HI sites of the expression vector pET25b (+) (Novagen) with the In-Fusion HD Cloning system (Clontech). The resultant plasmid, pET25-1348, was introduced into *E. coli* BL21 (DE3) (Merck) and the strain was grown at 37°C. The expression of the recombinant Os1348 protein was induced by the addition of isopropyl-β-D-thiogalactopyranoside at a final concentration of 0.4 mM and growth continued at 16°C for 16 h. Harvested cells were resuspended in PBS (pH 7.4) and lysed by sonication. Debris was removed by centrifugation at 40,000 × *g* for 30 min. The lysate was applied to a 5-ml HiTrap SP HP column (GE Healthcare). The column was washed with 20 mM sodium acetate (pH 5.2) and eluted with a linear gradient of 0–500 mM NaCl. Os1348 fractions were purified further on HiLoad 26/60 Superdex75 prep grade (GE Healthcare) pre-equilibrated with buffer consisting of 10 mM Tris–HCl (pH 7.5) and 150 mM NaCl. After dialysis at 4°C for 16 h against 20 mM sodium acetate (pH 5.2), the supernatant was purified by a Mono S 10/100 GL column (GE Healthcare) and a 0–300 mM NaCl elution gradient.

### X-ray Structural Elucidation of Os1348

The Os1348 protein was concentrated to 2.0 mg ml^–1^ and crystallized by the sitting-drop vapor diffusion method at 293 K using a precipitant solution consisting of 30% (w/v) polyethylene glycol monomethyl ester 2000 and 0.2 M MgCl_2_. Plate-type crystals with dimensions of 0.2 × 0.2 × 0.05 mm appeared within 2 weeks using 50 μl of the reservoir solution with a drop consisting of 0.5 μl of the protein solution and 0.5 μl of the reservoir solution.

Diffraction experiments for Os1348 crystals were conducted at the beamline BL-5A of Photon Factory (PF), High Energy Accelerator Research Organization, Tsukuba, Japan. Diffraction data were collected using the ADSC Q315 CCD detector (Area Detector Systems Corp., Poway, CA, United States). Crystals were mounted in a quartz glass capillary with a diameter of 0.3 mm (Capillary Tube Supplies Ltd., United Kingdom) and cryocooled in a nitrogen gas stream to 95 K. Crystals diffracted to 1.8 Å resolution. Data were integrated and scaled using the programs DENZO and SCALEPACK in the HKL2000 program suite (Otwinowski and Minor, 1997).

The crystal structure was elucidated by a direct method using the program AMPLE (Bibby et al., 2012) incorporated in the CCP4 program suite (Winn et al., 2011). Manual model building and molecular refinement were performed using Coot (Emsley and Cowtan, 2004) and Refmac5 (Murshudov et al., 2011). Data collection and refinement statistics are shown in Table 4. Model stereochemistry was assessed with the program Molprobity (Chen et al., 2010). Structural illustrations were drawn with the program PyMOL (DeLano, 2002).

### Detection of the Os1348 Protein

The presence of the Os1348 protein was examined in cells using western blotting with rabbit polyclonal antibodies raised against the recombinant Os1348 protein. The anti-Os1348 antibody was produced by Biogate (Gifu, Japan) according to their established protocol. Equal amounts of cells corresponding to 20 μl of a suspension with an OD_600 nm_ of 4.0 were collected. The pellet was solubilized in buffer using 4× Laemmli Sample Buffer (Bio-Rad) and boiled at 100°C for 5 min in a heating block. Samples were subjected to Tricine-SDS-PAGE (15% polyacrylamide gel) and subsequently analyzed by western blotting with the anti-Os1348 antibody.

### β-Galactosidase Assays

β-Galactosidase activity was quantified by the Miller method (Miller, 1972). *Pseudomonas* strains were grown at 28°C in 50-ml flasks containing 15 ml of NYB amended with 0.05% Triton X-100 with shaking at 180 rpm. Triton X-100 was required to prevent cell aggregation.

### Detection of Antibiotic Activity

The antibiotic activities of *Pseudomonas* Os17 and the mutant strains were assessed with *Bacillus subtilis* M168 (DSM 402, NBRC 111470) as the reporter. We also tested other *Pseudomonas* strains (*Pseudomonas syringae* pvs. *pisi* 730032 and *tabaci* 301612, *Pseudomonas chlororaphis* St508, and *P. protegens* Cab57) as the reporter to test susceptibility against *Pseudomonas* Os17. *P. syringae*, oomycete and fungal strains were obtained from the NARO Genebank in Tsukuba. In the assays with *B. subtilis* and other bacterial reporter strains, cultures of Os17 and the mutant strains were adjusted to OD_600 nm_ = 1.0 and 4-μl samples were spotted onto the modified GCM plate. After an overnight incubation at 28°C, cells were killed by UV irradiation on a transilluminator for 5 min. A soft-agar overlay for plates was prepared with 4-ml half-strength NA [2% (w/v) blood agar base, 0.25% (w/v) yeast extract] inoculated with 400-μl cultures of the reporter strains grown in NYB until the stationary phase. The overlay of *B. subtilis* revealed antibiotic production by growth inhibition zones. Halo sizes were measured from the edge of the bacterial spot to the end of the clear zone, and the data obtained are shown as the average of 3–6 replicates. In the assay with *Pythium ultimum* or *Fusarium oxysporum*, cultures of *Pseudomonas* strains were adjusted to OD_600 nm_ = 5.0 and 20-μl samples were streaked around the edge of the PDA plate, and, after the overnight incubation at 28°C, an inoculum of *P. ultimum* or *F. oxysporum* was transferred to the center of the plate. The plate was incubated at room temperature until *P. ultimum* or *F. oxysporum* reached its edge.

In the evaluation of DAPG production, strains were grown in modified GCM for 22 h after the inoculation (scaling up from an overnight culture to a fresh culture, 50 times, and grown in Erlenmeyer flasks at 180 rpm, 28°C), corresponding to an OD_600 nm_ of *ca.* 0.5. The supernatants of strains were extracted with an equal volume of ethyl acetate and analyzed by HPLC for DAPG as described previously (Schnider-Keel et al., 2000).

## Results

### Genomic Structure of the Putative Bacteriocin Gene Cluster in *Pseudomonas* sp. Strains Os17 and St29

**FIGURE 1:**
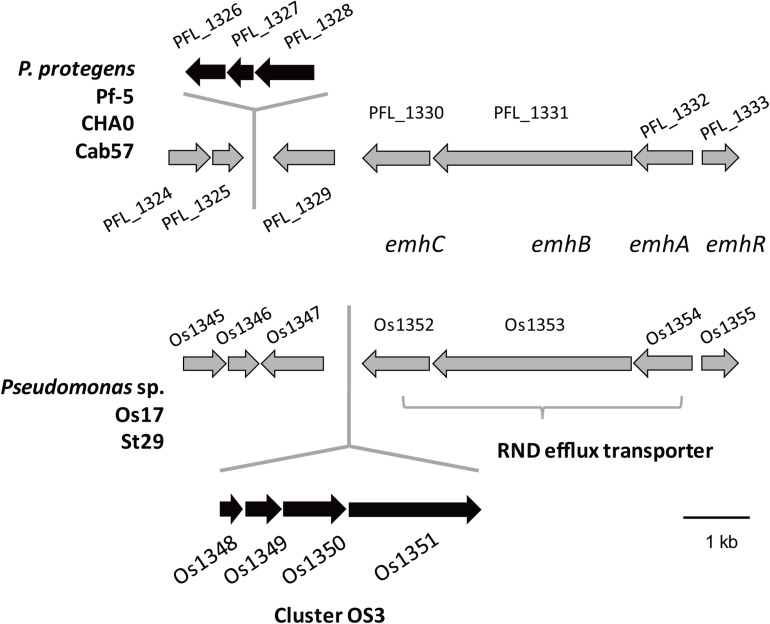
Physical map of the *Os1348* gene and surrounding genes in *Pseudomonas* sp. Os17 and St29 (lower) and corresponding regions in *P. protegens* Pf-5, CHA0, and Cab57 (upper). Gene IDs are given as those of Os17 and Pf-5. Common genes in the two groups are in gray, whereas genes that were not common are in black.

We previously identified 33 gene clusters that are shared by the genomes of Os17 and St29 and absent from the *P. protegens* Cab57 genome (Clusters OS1 to OS33 in Supplementary Table 4 of Takeuchi et al., 2015). Among these clusters, we focused on Cluster OS3, which was predicted to encode the bacteriocin gene cluster according to the antiSMASH program (Blin et al., 2013). In this program, the downstream genes (gene IDs were given as Os1352 and Os1353, predicted as transporter-related genes) were also included in this region as “bacteriocin.” The genomic structure of the region surrounding Cluster OS3 in *Pseudomonas* sp. Os17 (gene IDs were given as Os1348–Os1351) is shown in Figure 1 and the annotation of these ORFs is summarized in Table 1. The Os1348 protein was annotated as an alpha chain of nitrile hydratase. Although the actual function of the Os1348 protein as a nitrile hydratase has not yet been clarified, recent bioinformatic research that examined thiazole/oxazole-modified microcin (low-molecular-weight bacteriocins referred to as TOMM) precursors based on comparative genomics revealed a new class of post-translationally modified peptides with strong similarities to the alpha subunit of the nitrile hydratase (Haft et al., 2010). This finding, together with the annotation data of the downstream genes described below, prompted us to speculate that *Os1348* encodes the precursor and downstream genes are involved in post-translational modifications; however, conserved motifs among TOMM precursors, such as nitrile hydratase leader peptides, were not found in Os1348. Gene clusters encoding the predicted TOMM-type microcin have been identified in some pseudomonad genomes as homologs of the *mcb* operon that produces the DNA gyrase inhibitor microcin B17 (MccB17) in *E. coli* (Ghequire and De Mot, 2014). We also predicted the bacteriocin-encoding gene through the genomes of Os17 and St29 using the BAGEL 4 program (van Heel et al., 2018); however, Cluster OS3 was not detected, suggesting that it is not a typical bacteriocin. Only one gene in each strain (POS17_1251 for Os17 and PST29_1284 for St29) was predicted to be a putidacin by this program. We previously predicted that these two genes were the bacteriocin LlpA2 through annotation (Takeuchi et al., 2015). This gene was classified into 5,321 core genes that were shared with *P. protegens* Cab57.

**TABLE 1:** Similarities in *Pseudomonas* sp. Os17 ORFs to proteins in databases.

Gene ID of Os17*	Position	Products size (aa)	NCBI Ref. of the closest protein, blastP *E*-value	Predicted function	Number of Blast top hits**
**Cluster OS3 (1348-1351)**					
1348 (RS06725)	1495345.. 1495602	85	PMI18_04289 *Pseudomonas* sp. GM102 1.0E-29	Nitrile hydratase, alpha chain	2
			Pfam14407 5.2E-04	Frankia peptide, ribosomally synthesized peptide	
1349 (RS06730)	1495717.. 1496166	149	PMI18_04288 *Pseudomonas* sp. GM102 2.0E-50	Acetyltransferase	10
			Pfam00583 7.8E-10	Acetyltransferase (GNAT) family	
1350 (RS06735)	1496187.. 1497347	386	PMI18_04287 *Pseudomonas* sp. GM102 1.0E-133	Uncharacterized protein	4
			Pfam13485 1.5E-04	Peptidase MA superfamily (Gluzincin)	
			TIGR04267 9.3E-08	HEXXH motif domain	
1351 (RS06740)	1497363.. 1499315	650	PMI30_00628 *Pseudomonas* sp. GM50 0.0	SagB-type dehydrogenase domain-containing protein	6
			cd02142 7.6E-41	mcbC-like oxidoreductase	
**Putative transporter-related genes (1352–1354)**					
1352 (RS06745)	1499376.. 1500836 (complement)	486	YP_347010.1 *Pseudomonas fluorescens* Pf0-1 0.0	RND efflux system outer membrane lipoprotein NodT	41
			YP_258459.1, PFL_1330 *Pseudomonas protegens* Pf-5 0.0	Efflux transporter, outer membrane factor lipoprotein EmhC	
1353 (RS06750)	1500833.. 1503976 (complement)	1047	YP_258460.1, PFL_1331 *Pseudomonas protegens* Pf-5 0.0	Efflux transporter permease EmhB	>100
1354 (RS06755)	1503980.. 1505137 (complement)	385	YP_004352462.1 *Pseudomonas brassicacearum* subsp. *brassicacearum* NFM421 0.0	Acriflavine resistance protein A	>100
			YP_258461.1, PFL_1332 *Pseudomonas protegens* Pf-5 0.0	Efflux transporter, membrane fusion protein subunit EmhA	
1355 (RS06760)	1505403.. 1506053	216	ZP_05640495.1 *Pseudomonas syringae* pv. *tabaci* str. ATCC11528 4.0E-90	Transcriptional regulator TtgR	11
			YP_258462.1, PFL_1333 *Pseudomonas protegens* Pf-5 0.0	Transcriptional regulator EmhR	

*Gene ID given in the Pseudomonas genomic database (www.pseudomonas.com) are indicated in brackets. **The sequence identity cut-off was set to 90%. A Blast search was performed on July 23, 2018.

The downstream genes *Os1349–1351* were predicted to encode acetyltransferase, an uncharacterized protein (peptidase), and SagB-type dehydrogenase domain-containing protein, respectively, when predictions were performed using the Microbial Genome Annotation Pipeline (Takeuchi et al., 2015). A recent study on streptolysin, a bacteriocin from *Streptococcus pyogenes*, revealed that the 53-aa precursor peptide SagA was modified by the downstream gene products of *sagBCD* (homolog of the *mcb* operon), which encodes the dehydrogenase SagB, cyclodehydratase SagC, and docking protein SagD, respectively, jointly functioning for the introduction of thiazole and oxazole heterocycles onto SagA to be an active cytolysin (Lee et al., 2008). In the genome of Os17, the homologs of *sagA*, *sagC*, and *sagD* were not found in our search.

When we performed searches with protein BLAST, the N- and C-terminal regions of Os1350 showed similarities to the peptidase MA superfamily (Gluzincin, pfam13485) and HEXXH motif domain-containing protein (TIGR04267), respectively (Table 1). The Gluzincin family includes several zinc-dependent metallopeptidases and contains HEXXH and EXXXD motifs as part of their active site (NCBI; Hooper, 1994).

The genes *Os1348–1351* were not as commonly conserved in other strains as the downstream genes (*Os1352-1355*) based on the number of top hits of each gene in the Blastp analysis (Table 1). None of the three *P. protegens* strains tested (Cab57, Pf-5, and CHA0) possessed the genes *Os1348–1351*, whereas they possessed the homologs of *Os1352-1355* (Figure 1). The *Os1352-1354* gene cluster was predicted to encode the EmhABC efflux transporter, and Os1355 was annotated as their transcriptional regulator EmhR. The homolog of this region has been characterized in the DAPG-producing biocontrol strain *Pseudomonas fluorescens* 2P24, in which the deletion of each gene of *emhABC* increased the extracellular accumulation of DAPG, whereas the *emhR-*deficient mutant decreased the biosynthesis of DAPG (Tian et al., 2010).

### Construction of Os1348 and Downstream Gene Mutants and Their Effects on Antibiotic Activity

**FIGURE 2:**
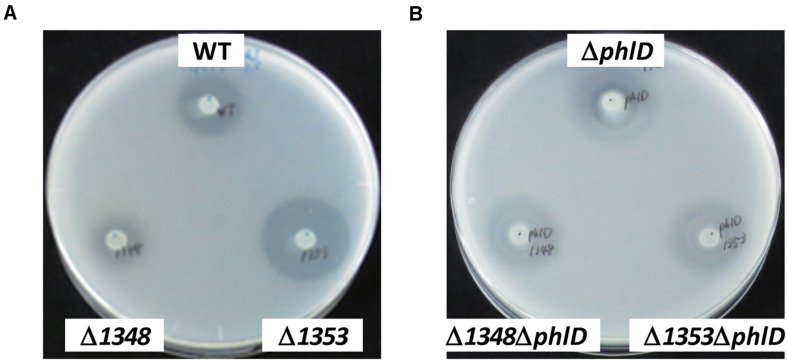
Effects of the *phlD* mutation on antibiotic activity. The antibiotic activities of *Pseudomonas* strains grown on modified GCM plates were evaluated by the size of the growth inhibition zone of *B. subtilis.* **(A)** The antibiotic activities of the *Pseudomonas* sp. Os17 wild-type (WT), *1348* mutant (Δ*1348*), and *1353* mutant (Δ*1353*) were compared. **(B)** The effects of the *phlD* mutation on each strain in panel **(A)** were compared as follows: *phlD* mutant (Δ*phlD*), *1348*/*phlD* mutant (Δ*1348*Δ*phlD*), and *1353*/*phlD* mutant (Δ*1353*Δ*phlD*) were compared.

Annotation data prompted us to predict that this region contributes to the antibiotic activity of the strains that possess it. We investigated whether the deletion of each gene affected the antibiotic activity of strain Os17 in a biotest using *B. subtilis*, which has been used as an indicator strain to test susceptibility against pseudomonads (Takeuchi et al., 2009, 2012, 2014, 2015), on modified GCM plates. The rationale for using this medium was to prevent the excessive halo size observed in strain Os17. Growth inhibitory activity against *B. subtilis* was weaker in the *Os1348* mutant than in the wild-type (Table 2 and Figure 2). Based on the well-known function of bacteriocins, in which they are only deleterious to members of a certain bacterial species or a subset of phylogenetically close relatives of the producer, we also tested other *Pseudomonas* strains (*P. syringae* pvs. *pisi* 730032 and *tabaci* 301612, *P. chlororaphis* St508 and *P. protegens* Cab57) as the reporter of close relatives (Supplementary Figure 2). *P. syringae* and *P. chlororaphis* strains were sensitive to the Os17 wild-type showing a dim halo; however, the Os1348 mutant exhibited similar inhibitory activity to the wild-type, and thus, the effect of Os1348 on sensitivity was not applicable to these strains. Regarding both pathovars of *P. syringae*, Os17 strains spotted onto the plate exhibited a swarm pattern for an unknown reason. We tested *P. protegens* Cab57 as the reporter of a closer relative, and found that it was resistant to strain Os17. In a biotest using the phytopathogenic oomycete *P. ultimum* and the fungus *Fusarium oxysporum*, the weaker antibiotic activity of the *Os1348* mutant was not observed (data not shown).

**TABLE 2:** Effects of mutations in each gene on antibiotic activity.

Test number and bacterial strain	Halo size against *Bacillus subtilis* (mm)^*a*^
1	
Os17 (wild-type)	7.0 ± 1.3
Δ*1348*	5.0 ± 0.6*
2	
Os17	8.0 ± 0.0
Δ*1349*	8.8 ± 1.2
3	
Os17	8.7 ± 0.9
Δ*1350*	8.3 ± 1.1
4	
Os17	6.8 ± 0.7
Δ*1351*	3.5 ± 0.4*
5	
Os17	6.5 ± 0.4
Δ*1353*	10.0 ± 0.0*
6	
Os17	6.8 ± 0.3
Δ*1348* + *1348*	6.9 ± 0.2
7	
Os17	8.1 ± 0.6
Δ*gacA*	ND

^*a*^Data are shown as the averages of 3–6 replicates ± standard deviation. Halo sizes were measured from the edge of the bacterial spot to the end of the clear zone. *Indicates a significant difference from that in Os17 at *P* < 0.05 (*t-*test).

The deletion of *Os1351* also reduced antibiotic activity against *B. subtilis*, whereas the other two (*Os1349-* and *Os1350-*) mutants did not exhibit weaker antibiotic activity (Table 2). All mutants grew at similar rates to the wild-type in the same liquid medium, except for the *gacA* mutant, which showed cell aggregation in the stationary phase of the growth curve. This autoaggregation may be the reason for the reduced OD_600_ (Supplementary Figure 1).

We also investigated the effects of a mutation in the putative efflux transporter gene *Os1353* (the *EmhB* homolog) on antibiotic activity. The *Os1353* mutant exhibited stronger antibiotic activity than the wild-type (Table 2). This result is consistent with the phenotype of the *emhB* mutant in strain *P. fluorescens* 2P24, which showed increased DAPG production levels (Tian et al., 2010). The deduced aa sequence of EmhB in *P. fluorescens* 2P24 shared 89% identity with the *Os1353* gene product. An increased level of extracellular DAPG production was observed in the *Os1353* mutant (Table 3), suggesting that stronger antibiotic activity by the *Os1353* mutant was due, at least in part, to a higher production level of DAPG. This increase was abolished by an additional mutation in the *phlD* gene, which encodes polyketide synthase, a key enzyme in DAPG biosynthesis (Table 3). The deletion in the *Os1348* gene did not affect the production level of DAPG under the conditions tested. The additional mutation in the *phlD* gene on the *Os1348* and *Os1353* mutants also abolished the clear halo, whereas the single mutant of *phlD* did not (Figure 2), suggesting that the antibiotic activity observed in this experiment mainly accounts for the DAPG production level of each strain, and that *Os1348* and *Os1353* are involved in the expression of antibiotic activity other than that caused by DAPG. The hazy halo observed in the *1348/phlD* double mutants (and also in *1353/phlD*) may have been due to the production of remaining secondary metabolites. This will be addressed in the section “Discussion.”

**TABLE 3:** DAPG production levels by *Pseudomonas* sp. Os17 and its mutants grown in modified GCM medium.

Bacterial strain tested	[DAPG] (nmol OD_600_^–1^)^*a*^
Os17 (wild-type)	1.07 ± 0.18
Δ*1348*	1.87 ± 0.91
Δ*1353*	49.86 ± 15.31*
Δ*phlD*	ND
Δ*1348*Δ*phlD*	ND
Δ*1353*Δ*phlD*	ND
Δ*gacA*	ND

^*a*^Data are shown as the averages of three replicates ± standard deviation. DAPG concentrations were indicated per optical density at 600 nm (OD_600_) in strains grown in modified GCM to OD_600_ = 0.5 (one OD_600_ unit corresponds to approximately 1 × 10^9^ cells/ml). *Indicates a significant difference from that in Os17 at *P* < 0.01 (*t-*test).

### Expression of the Recombinant Os1348 Protein in *E. coli*

To obtain a more detailed understanding of the characteristics and structure of Os1348, we generated a full-length recombinant Os1348 protein without the use of tags by expression in *E. coli*.

The antibiotic activity of the recombinant Os1348 protein toward *B. subtilis* was tested, but not detected (data not shown), suggesting that the Os1348 protein needs to be modified post-translationally in order to exert its antibiotic effects or that the protein itself does not act as an antimicrobial protein that directly inhibits the growth of *B. subtilis*.

### Downstream Genes of Os1348 Affect the Affinity of the Os1348 Protein Toward the Antibody

**FIGURE 3:**
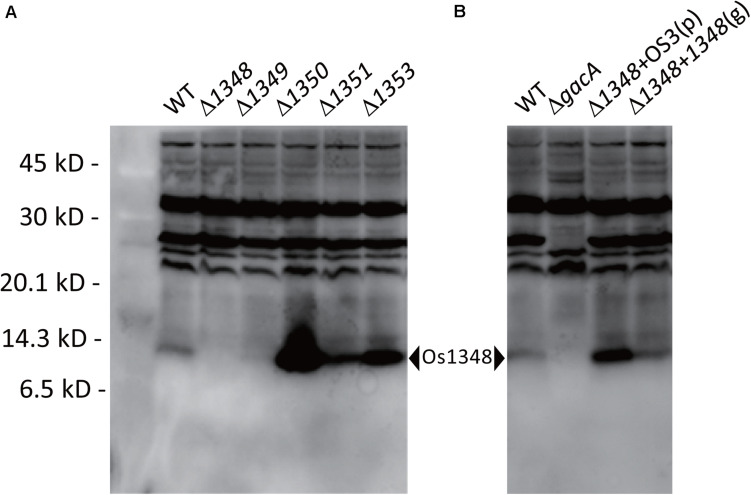
Detection of the Os1348 protein in whole-cell lysates of *Pseudomonas* sp. Os17 and mutant strains using western blotting with rabbit polyclonal antibodies raised against the recombinant Os1348 protein. **(A)** Detection of the Os1348 protein in the *Pseudomonas* sp. Os17 wild-type (WT), Cluster OS3 (*1348-1351*) mutants, and the *1353* mutant derived from it. **(B)** Detection of the Os1348 protein in the *Pseudomonas* sp. Os17 wild-type (WT), *gacA* mutant (Δ*gacA*), complemented *1348* mutant with the plasmid pME6031-OS3 [Δ*1348* + OS3(p)], and complemented *1348* mutant by the genome in its original position [Δ*1348* + *1348*(g)]. Whole-cell extracts were prepared from strain *Pseudomonas* sp. Os17 and the mutants grown in GCM 18 h after the inoculation (scaling up from an overnight culture to a fresh culture, 100 times, and grown in Erlenmeyer flasks at 180 rpm, 28°C). Equal amounts of cells corresponding to 20 μl of a suspension with an OD_600__nm_ of 4.0 were collected. The pellet was solubilized in sample buffer and then subjected to Tricine-SDS-PAGE (15% polyacrylamide gel).

We hypothesized that the Os1348 protein was modified post-translationally. Therefore, we assessed the Os1348 protein status in *Pseudomonas* sp. Os17. By utilizing rabbit polyclonal antibodies raised against the Os1348 recombinant protein, we detected the Os1348 protein (*ca.* 8.9 kDa) with western blotting in wild-type cells, but not in *Os1348* mutant cells. Intracellular levels of the *Os1348* protein differed in *Os1350-* or *Os1351-*disrupted cells at higher levels of detection and in *Os1349-* disrupted cells at lower levels of detection from that in wild-type cells, suggesting that the Os1348 protein levels in these mutants are quantitatively different from that in the wild-type or that the expression level of the Os1348 protein is under the control of downstream genes (Figure 3). The increased level of the Os1348 protein in the *1350* mutant is consistent with the predicted function of the Os1350 protein as a peptidase; however, peptidase activity has not yet been demonstrated. The mobility shift in molecular mass was not observed in the *1351* mutant, suggesting that it has little, if any, responsibility for modifications. Assuming that the Os1351 protein functions as a dehydrogenase, an increase in multiples of 2 Da will be observed in the deficient mutant, which may have been difficult to detect using the present method.

We also subjected the supernatant of the culture medium of the wild-type to western blotting to investigate the status of protein secretion into the medium. However, it was below the detection limit (data not shown).

### Effects of Complementation of the *1348* Mutant

Complementation of the *1348* gene into the chromosome at its original position restored antibiotic activity (Table 2) as well as the expression level of the Os1348 protein (Figure 3) to wild-type levels, indicating that the phenotype was due to inactivation of the *1348* gene and not to polar effects on neighboring genes. Regarding the complementation of the *1348* mutant by a plasmid, we constructed a plasmid harboring *1348-1351* genes (full region of OS3) based on the potential for post-translational modifications by the products of the downstream genes of *1348*. Complementation of the *1348* mutant by this plasmid led to higher expression levels of Os1348 than that of the wild-type, presumably because a multicopy plasmid was used (Figure 3). However, complementation by plasmids did not restore antibiotic activity (data not shown), suggesting that the gene needs to be located in the original position to exert its function on antibiotic activity, i.e., the OS3 cluster needs to be located upstream of the putative transporter-related genes (*Os1352*-*1354*) in order for the Os1348 protein to be exported. In a number of bacteriocins, the genetic organization of bacteriocin gene cluster have been found to include transporter genes in the downstream region (Haft et al., 2010; Ghequire and De Mot, 2014), suggesting that the positioning of transporter genes is relevant.

### X-ray Crystal Structure of Os1348

**FIGURE 4:**
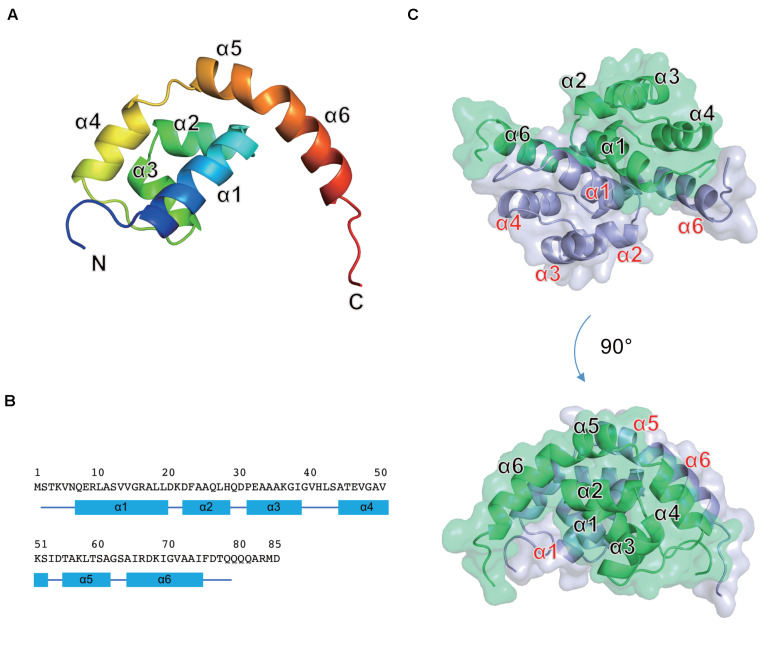
Crystal structure of the Os1348 protein. **(A)** The monomeric structure is shown as a ribbon diagram and colored in the rainbow mode from the N terminus (blue) to C terminus (red). **(B)** Amino acid sequence and secondary structure. Secondary structure elements are shown below the sequence. α-Helices and loops are drawn as quadrilaterals and lines, respectively. **(C)** The homodimeric structure is shown in ribbon and transparent surface representations. Individual monomers are shown in light blue and light green, respectively.

The full-length Os1348 protein was overexpressed in *E. coli*, purified, and crystallized. The Os1348 structure was elucidated by the molecular replacement method using the ideal helices mode at a resolution of 1.9 Å (Figure 4 and Table 4). The final model included four Os1348 molecules consisting of 2–79 residues in the asymmetric unit. The six C-terminal residues were not observed in the electron density map and appeared to be highly flexible.

**TABLE 4:** X-ray diffraction data collection and structural refinement statistics.

Data collection statistics	
Beamline	PF-BL-5A
Wavelength (Å)	1.000
Space group	*P*2_1_
Unit cell (Å)	*a* = 58.6, *b* = 33.6, *c* = 66.2, β = 92.8°
Resolution (Å)^a^	100.0–1.90 (1.97–1.90)
Number of refractions	
Observed	130,688
Unique^a^	20,568 (2,065)
Completeness (%)^a^	99.8 (99.7)
Multiplicity^a^	3.3 (3.0)
I/σ(I)^a^	9.3 (2.6)
R_*merge*_^a^	0.154 (0.645)
Refinement statistics	
Resolution (Å)^a^	44.96–1.90 (1.95–1.90)
R-factor^a^	0.170 (0.222)
Free R-factor^a^	0.224 (0.312)
Number of protein atoms	2,109
Number of waters	217
Rms deviations from ideality	
Bond lengths (Å)	0.010
Bond angles (°)	1.549
Ramachandran plot	
Preferred regions (%)	97.5
Allowed regions (%)	2.5
Outliers (%)	0

*^*a*^Values for the highest resolution shell are given in parentheses.*

The Os1348 monomer was composed of six α helices (α1-α6) and formed a Magatama (comma)-shaped α-structure (Figures 4A,B). Two Os1348 monomers joined and formed a dimeric structure. The Os1348 dimer had a semicircular shape (Figure 4C). The dimer interface was composed of many hydrophobic amino acids from the α1, α4, α5, and α6 helices. Hydrophilic interactions, namely, hydrogen bonds, and salt bridges, also appeared to contribute to the stability and solubility of the Os1348 dimer. Based on its three-dimensional structure, the free energy values ΔG_*int*_ and ΔG_*diss*_ of the Os1348 dimer were calculated by the PISA website as −20.4 and 17.0 kcal/mol, respectively, suggesting that the Os1348 protein formed a stable homodimer in solution.

The Os1348 protein showed no structural similarities to other bacteriocins or proteins registered in the PDB. Although the Os1348 protein was annotated as an alpha chain of nitrile hydratase, no structural similarities to the nitrile hydratases registered in the PDB were observed. Therefore, the function of the Os1348 protein and mechanisms underlying its antimicrobial activity remain unclear. Based on the results of biological investigations, the full-length Os1348 protein may be a bacteriocin precursor and appears to be post-translationally modified into an active form by the products of downstream genes.

### The *gacA* Mutant Exhibits Decreased Expression Levels of *Os1348′-′lacZ*

In *P. protegens* and other fluorescent pseudomonads, the GacS/GacA system positively controls secondary metabolism. We previously reported that the *gacA* mutant of strain Os17 lost its antibiotic activity against *B*. *subtilis*, similar to other biocontrol strains of pseudomonads. Furthermore, the production of rhizoxin analogs, which were found to be major biocontrol factors of this strain, was also under the control of GacA (Takeuchi et al., 2015). To establish whether the production of the Os1348 protein in strain OS17 was also under the control of the GacS/GacA system, we examined production levels using western blotting. In the *gacA* mutant, the accumulation of the Os1348 protein was below the detection limit (Figure 3).

**FIGURE 5:**
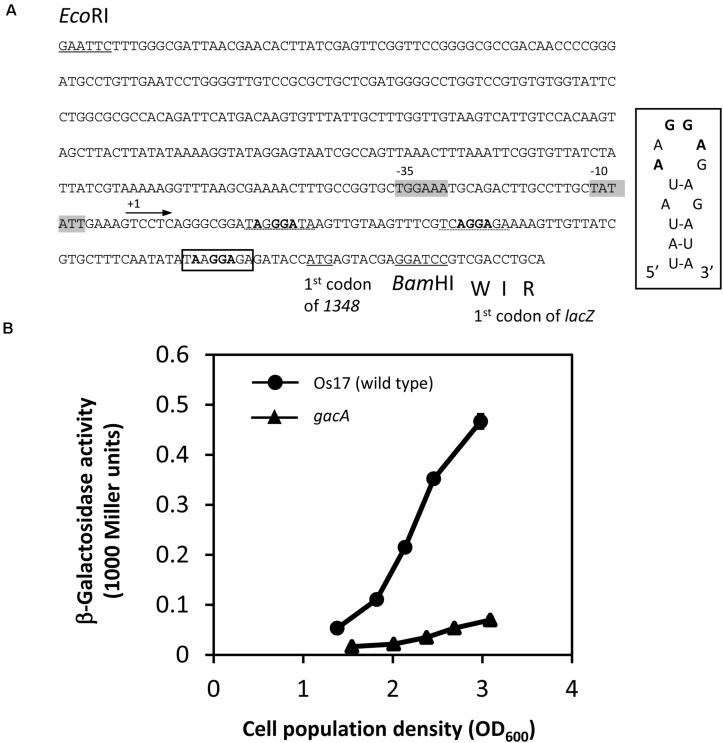
Construction and expression of the *1348′-′lacZ* fusion on pME6014-1348 in *Pseudomonas* sp. Os17 and the *gacA* mutant. **(A)** A 420-bp *Eco*RI/*Bam*HI fragment from the upstream region of *Os1348* containing the first three codons of Os1348 was fused to the first codon of *lacZ* in the vector pME6014, with an interval of the *Bam*HI site in frame, to give pME6014-1348. A putative RsmA/E binding site is shown in a box upstream of the predicted ATG start codon of *Os1348*. Inset, predicted secondary structure of the *Os1348* mRNA leader sequence near the translation start site when complexed with RsmA/E. The typical GGA motif is shown in bold face. Other candidates of the GGA motif are shown with the dashed lines. The putative –35 and –10 promoter sequences are shown in gray boxes. **(B)** Expression of a *1348′-′lacZ* fusion on pME6014-1348 in *Pseudomonas* sp. Os17 and the *gacA* mutant grown in nutrient yeast broth (NYB). The symbols indicate the averages of triplicate cultures and bars indicate standard deviations. OD_600__nm_, optical density at 600 nm.

In Cluster OS3 of the Os17 genome, the *Os1348* gene has a potential RsmA/E binding site (UAAGGAGA) in its upstream region, which may expose the conserved **A**A**GGA**G hexa-loop on a U-A base pair (underlined). Therefore, we constructed translational *Os1348′-′lacZ* fusion, and compared expression levels in the wild-type and *gacA* mutant (Figure 5). In the *gacA* mutant, a decreased level of expression was observed at high cell densities, suggesting that the GacA-dependent regulation of the gene may account for the decreased level of the Os1348 protein in the *gacA* mutant.

## Discussion

The present results demonstrated that comparative genomics among closely related root-colonizing pseudomonad strains, including model and non-model strains, revealed a novel gene cluster whose expression contributes to the antibacterial activity of the non-model strain. Since the genomic sequence of model strain Pf-5 was completely elucidated (Paulsen et al., 2005), it has provided novel insights into genome-mining strategies to identify antimicrobial secondary metabolites from agriculturally important bacteria (Gross et al., 2007). These strategies have also identified small proteinaceous and peptidic structures, such as bacteriocins, which are ribosomally synthesized and post-translationally modified (Ghequire and De Mot, 2014). The relatively large genome sizes of *P. protegens* (*ca.* 6.7–7.1 Mbp^1^) and related strains are attributed to the variety of genomic repertoires for the synthesis of competitive weapons against other organisms in the rhizosphere. The genome sizes of our recently isolated strains Os17 and St29, which were revealed to be same species, were 6.89 and 6.83 Mbp, respectively (Takeuchi et al., 2015). Some clusters conserved in both strains were predicted to be involved in the production of secondary metabolites, and one, named Cluster OS3, was examined in the present study.

In the genome of strain Os17, there are several gene clusters other than *phl* (for the production of DAPG) that are involved in the biosynthesis of known secondary metabolites with antibiotic activities. The representative clusters are *rzx* and *hcn* (for the production of rhizoxin analogs and HCN, respectively). The *aprA* gene cluster, for the major extracellular protease AprA, is also conserved in this strain (Takeuchi et al., 2015). Under the experimental conditions tested in the present study, DAPG is the major factor for inhibitory activity of *B. subtilis*, and the minor is related to the cluster OS3. The hazy halo observed in the *1348/phlD* double mutant (and also in *1353/phlD*) in Figure 2 may due to the production of remaining secondary metabolites. It is important to note that reciprocal regulation between antibiotics has been reported in *P. protegens* strains CHA0 and Pf-5, while that of pyoluteorin and the phloroglucinol derivatives has been investigated in the most detail (Schnider-Keel et al., 2000; Brodhagen et al., 2004; Baehler et al., 2005; Kidarsa et al., 2011). In the present study, the mutational loss of the *phlD* gene may have affected the production of antibiotics other than pyoluteorin, which is absent from Os17, and, thus, resulted in the hazy halo.

Two gene clusters, *plt* and *prn*, which are involved in the production of pyoluteorin (PLT) and pyrrolnitrin (PRN), respectively, were found to be missing in the genomes of strains Os17 and St29. They are two out of four typical gene clusters of *P. protegens* other than *phl* and *hcn*. Genomic variations may contribute to the uniqueness of the species of these strains. The size of strains Os17 and St29 were equivalent to that of *P. protegens* strains; this may be due to the presence of unknown gene clusters that encode novel secondary metabolites. For example, in the genome of strain Os17, two neighboring clusters were partly found to be homologs of the biosynthetic clusters for the antibiotic mitomycin from *Streptomyces lavendulae* (Takeuchi et al., 2015). Although the function of these clusters has not yet been elucidated, they will contribute to the identification of novel secondary metabolites.

In the present study, a functional analysis of the gene cluster *Os1348–Os1351* was performed, and the *Os1349–Os1351* genes were predicted to be involved in post-translational modifications to the small precursor protein Os1348. The deletion of *Os1350* resulted in an increased Os1348 protein level (Figure 3). This result is consistent with the predicted function of the Os1350 protein as a peptidase, in which the N-terminal region of Os1350 showed similarities to the peptidase MA superfamily (Gluzincin); however, peptidase activity has not yet been demonstrated. Peptidases and proteases need to have strict selectivity in order to avoid the deleterious and random degradation of functional proteins. A recent study on a metallopeptidase-harboring gluzincin motif revealed that it recognized *O*-linked glycan modifications on protein substrates for its catalytic machinery (Noach et al., 2017). If the Os1350 protein functions as a peptidase and targets the Os1348 protein, this raises a number of questions about selectivity, i.e., whether post-translationally modified proteins are recognized. There is another speculation on the increased level of Os1348 observed in the *1350* mutant, concerning the inability of the cell to export the unmodified peptide.

Regarding *Os1351*, which was predicted to encode a SagB-type dehydrogenase domain-containing protein, an important finding was previously reported on streptolysin, a bacteriocin from *S. pyogenes*, as described above. Besides streptolysin, a similar mechanism of modification is utilized in the biosynthetic pathways for microcin B17 (Ghequire and De Mot, 2014), the patellamides (Schmidt et al., 2005), and the thiopeptides (Wieland Brown et al., 2009). The products of these clusters have been collectively classified as TOMMs due to the conservation of their genetic and chemical structures (Haft et al., 2010). Although the relevance of Sag-type modifications remains unknown in Os17, a more detailed understanding of the roles and mechanisms underlying protein modifications using gene products of the OS3 cluster represents a future challenge in bioengineering.

The Os1348 protein in the present study cannot be regarded as a bacteriocin from a rigorous definition point of view because we were unable to demonstrate that it was secreted into the medium due to the detection limit. The mutational loss of the putative efflux transporter gene *Os1353* increased the protein accumulation of Os1348 in the cell, which suggests the presence of a transport system. Considering the spectrum of the strains that were tested, Os1348 may not be categorized as a typical bacteriocin, which is defined as toxins against those that are closely related to bacteriocin-producing strains.

In addition to direct antibiotic activity, peptides secreted through a bacteriocin export system have been shown to possess signaling functions in a manner of quorum sensing and bacterial crosstalk (Michiels et al., 2001). The Os1348 protein also showed homology to the Type VI secretion protein ImpH (27% identical at 41 aa without gaps, Supplementary Figure 3), which was originally identified as one of the gene products of the nodulation impairment locus *imp* in *Rhizobium leguminosarum* (Roest et al., 1997; Bladergroen et al., 2003). Since the Type VI secretion system (T6SS) has been shown to play various roles in the social behavior of Gram-negative bacteria, besides the primary role assigned to T6SS as a potent weapon during interbacterial competition, investigations on the possible functions of the Os1348 protein as a signaling molecule will be an interesting aspect of future studies. Omics approaches, such as RNA-seq analyses based on comparisons of wild-type and mutant strains, may provide a more accurate view of the possible function of this protein in a signaling role.

In the present study, we did not detect the antibiotic activity of the full-length recombinant Os1348 protein, the crystal structure of which showed no structural similarities to other bacteriocins or proteins registered in the PDB. The native Os1348 protein is suggested to be post-translationally modified by the products of downstream genes, and this maturation process may be indispensable for this protein to be an active form. Further studies are needed to investigate whether the native Os1348 protein forms a dimer, and if so, whether the dimerization of the Os1348 protein affects the biological activity of this protein. Taken together with the relevance of post-translational modifications in this protein, a structural analysis of the native Os1348 mature protein combined with that of each mutant of the downstream genes will provide an in-depth quality assessment of the protein.

## Data Availability Statement

The datasets presented in this study can be found in online repositories. The names of the repository/repositories and accession number(s) can be found below: https://doi.org/10.2210/pdb6LK7/pdb.

## Author Contributions

KT and WT conceived and designed research. KT, WT, ZF, and KY conducted the experiments. NS contributed the strains. KT, WT, ZF, and TY analyzed the data. KT, WT, and ZF wrote the manuscript. All authors read and approved the manuscript.

## Conflict of Interest

The authors declare that the research was conducted in the absence of any commercial or financial relationships that could be construed as a potential conflict of interest.

## Footnotes

1 www.pseudomonas.com
