# Supplementary material for: Discovery of an Antibiotic-Related Small Protein of Biocontrol Strain Pseudomonas sp. Os17 by a Genome-Mining Strategy
Source: Front Microbiol. 2020 Nov 26;11:605705. doi: 10.3389/fmicb.2020.605705 (PMC7726476; doi:10.3389/fmicb.2020.605705)
Supplement: Supplementary file 1 [file Presentation_1.pptx]

## Slide 1
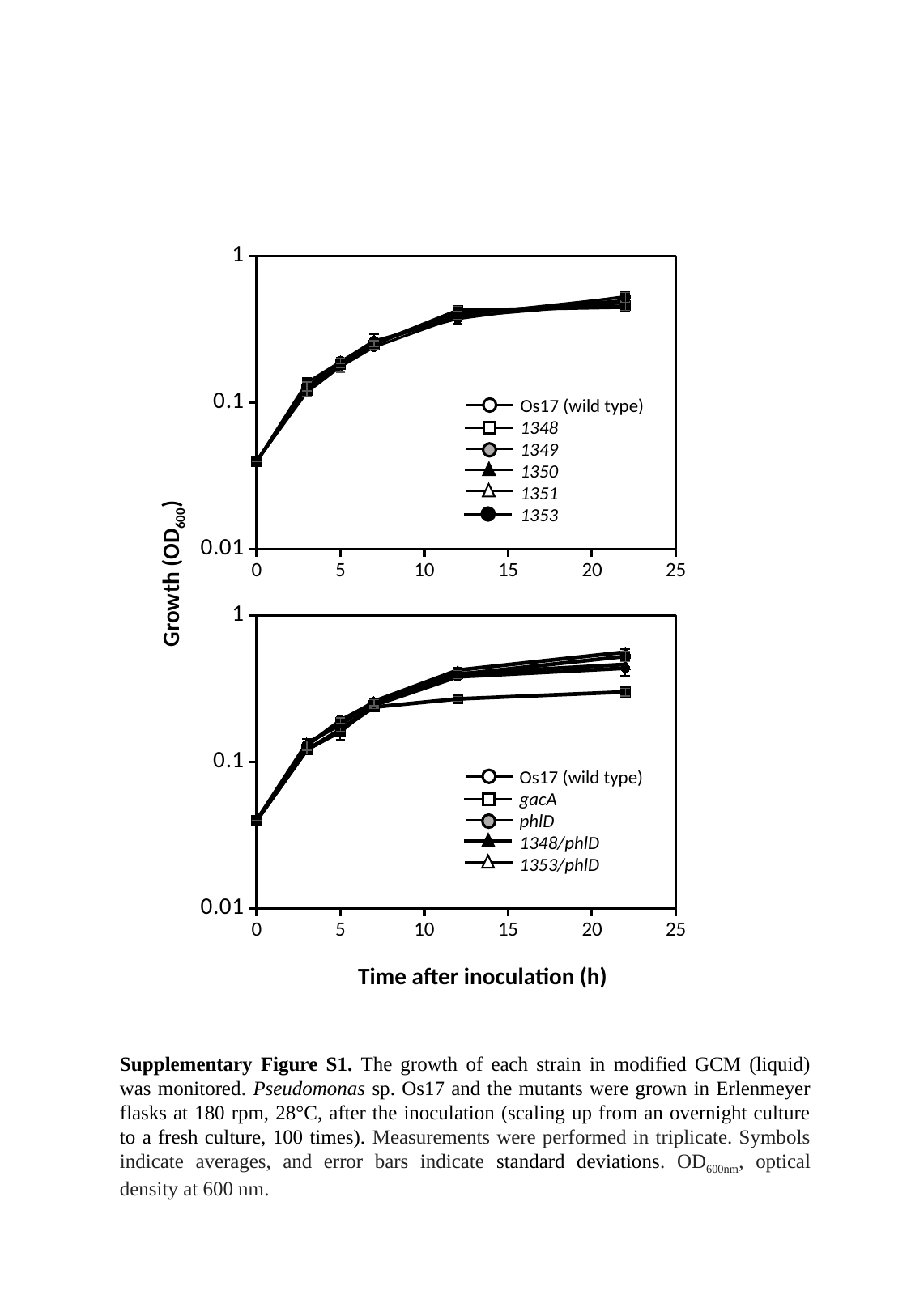

### Chart
| Category | | | | | | |
|---|---|---|---|---|---|---|Os17 (wild type)
1348
1349
1350
1351
1353
Growth (OD600)
### Chart
| Category | | | | | |
|---|---|---|---|---|---|Os17 (wild type)
gacA
phlD
1348/phlD
1353/phlD
Time after inoculation (h)
Supplementary Figure S1. The growth of each strain in modified GCM (liquid) was monitored. Pseudomonas sp. Os17 and the mutants were grown in Erlenmeyer flasks at 180 rpm, 28°C, after the inoculation (scaling up from an overnight culture to a fresh culture, 100 times). Measurements were performed in triplicate. Symbols indicate averages, and error bars indicate standard deviations. OD600nm, optical density at 600 nm.

## Slide 2
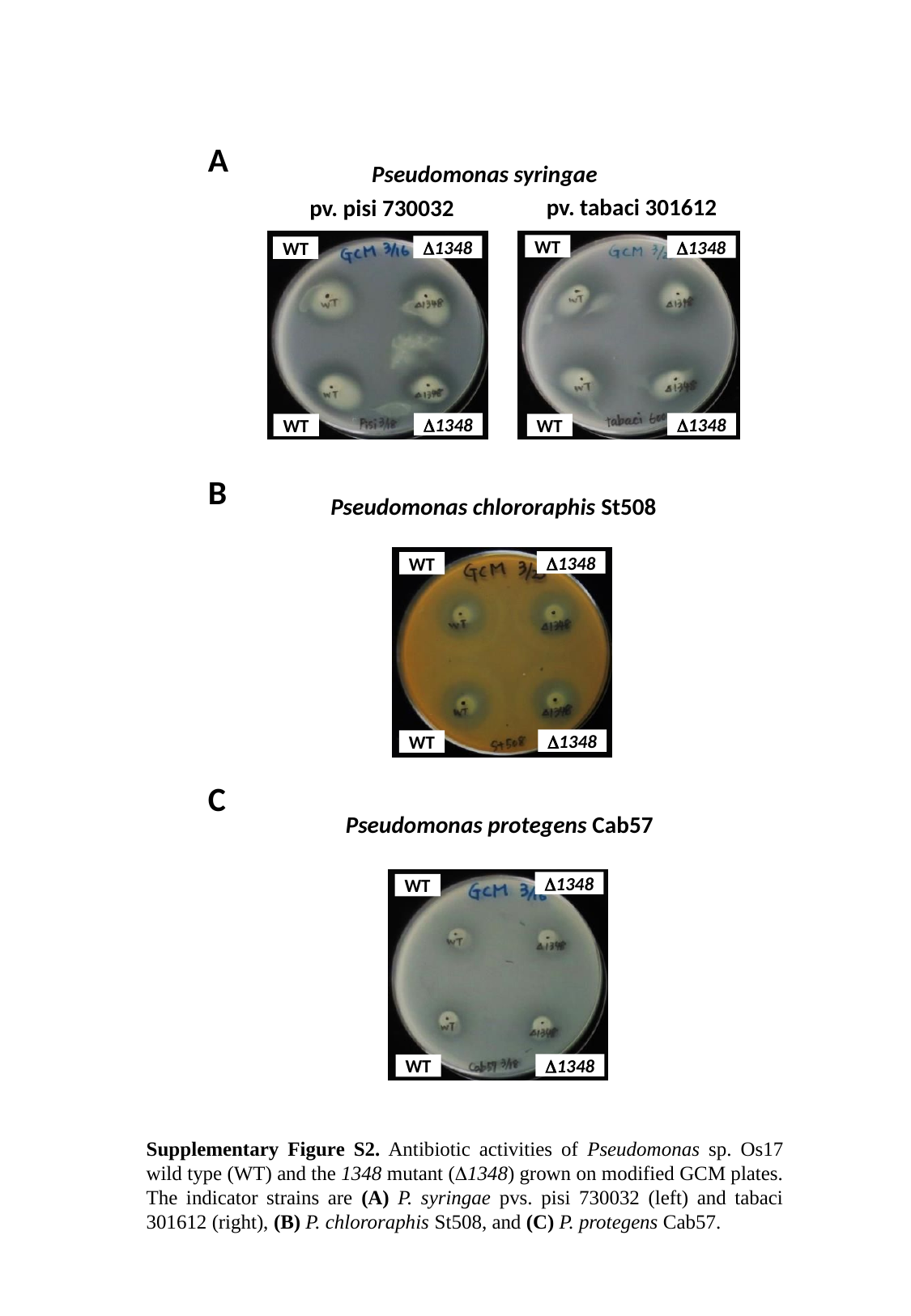

A
Pseudomonas syringae
pv. tabaci 301612
pv. pisi 730032
WT
D1348
D1348
WT
D1348
D1348
WT
WT
B
Pseudomonas chlororaphis St508
D1348
WT
D1348
WT
C
Pseudomonas protegens Cab57
D1348
WT
D1348
WT
Supplementary Figure S2. Antibiotic activities of Pseudomonas sp. Os17 wild type (WT) and the 1348 mutant (Δ1348) grown on modified GCM plates. The indicator strains are (A) P. syringae pvs. pisi 730032 (left) and tabaci 301612 (right), (B) P. chlororaphis St508, and (C) P. protegens Cab57.

## Slide 3
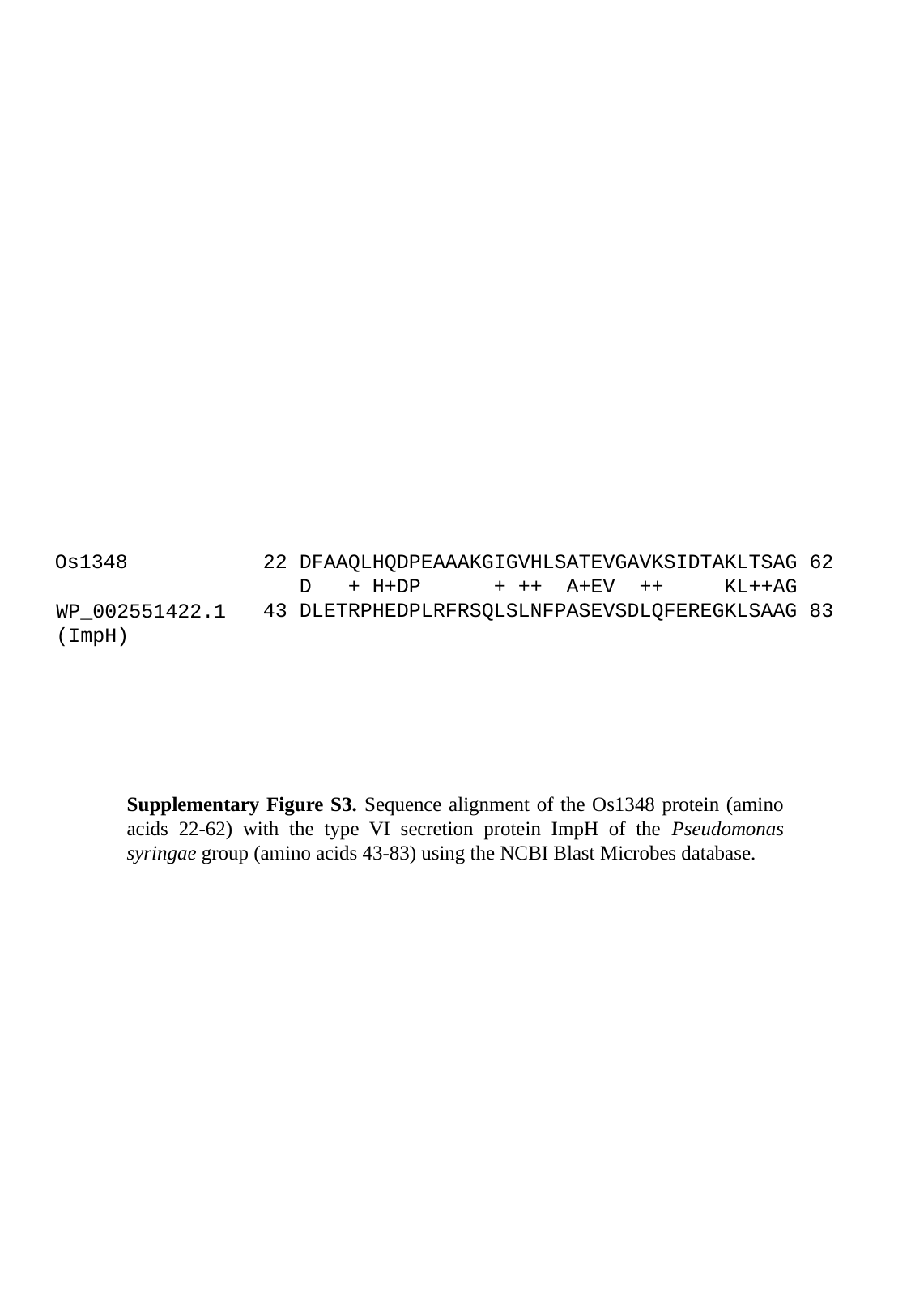

Os1348
22 DFAAQLHQDPEAAAKGIGVHLSATEVGAVKSIDTAKLTSAG 62
 D + H+DP + ++ A+EV ++ KL++AG
43 DLETRPHEDPLRFRSQLSLNFPASEVSDLQFEREGKLSAAG 83
WP_002551422.1
(ImpH)
Supplementary Figure S3. Sequence alignment of the Os1348 protein (amino acids 22-62) with the type VI secretion protein ImpH of the Pseudomonas syringae group (amino acids 43-83) using the NCBI Blast Microbes database.
