# Supplementary material for: Discovery of an Antibiotic-Related Small Protein of Biocontrol Strain Pseudomonas sp. Os17 by a Genome-Mining Strategy
Source: Front Microbiol. 2020 Nov 26;11:605705. doi: 10.3389/fmicb.2020.605705 (PMC7726476; doi:10.3389/fmicb.2020.605705)
Supplement: Supplementary file 2 [file Data_Sheet_1.docx]

Supplementary Material

**Supplementary Tables**

**Supplementary Table S1**

Bacterial strains and plasmids used in the present study

| Strain or plasmid | Description | Source or reference |
| --- | --- | --- |
| Strains | |  |
| *Bacillus subtilis* | |  |
| M168 | Wild type | DSM, NBRC |
| *Escherichia coli* | |  |
| DH5α, HB101 | Laboratory strains | Sambrook et al. 2001 |
| *Pseudomonas* sp. | |  |
| Os17 | Wild type | NARO Genebank |
| Os17gacA | Δ*gacA* | Takeuchi et al. 2015 |
| Os17phlD | Δ*phlD* | This study |
| Os17-1348 | Δ*1348* | This study |
| Os17-1348phlD | Δ*1348*Δ*phlD* | This study |
| Os17-1348C | Δ*1348+1348*, Δ*1348* strain with the 1348 region in its original genomic position | This study |
| Os17-1349 | Δ*1349* | This study |
| Os17-1350 | Δ*1350* | This study |
| Os17-1351 | Δ*1351* | This study |
| Os17-1353 | Δ*1353* | This study |
| Os17-1353phlD | Δ*1353*Δ*phlD* | This study |
| *Pseudomonas chlororaphis* St508 | | Morohoshi et al. 2017 |
| *Pseudomonas protegens* Cab57 | | NARO Genebank |
| *Pseudomonas syringae* pv. pisi 730032 | | NARO Genebank |
| *Pseudomonas syringae* pv. tabaci 301612 | | NARO Genebank |
|  | | |
| Plasmids |  |  |
| pCR-Blunt II-TOPO | Cloning vector, pUC *ori*; Km^r^ | Invitrogen |
| pET25b (+) | Inducible expression vector, Ap^r^ | Novagen |
| pET25-1348 | pET25b (+) containing a *1348* region , Ap^r^ | This study |
| pME497 | Mobilizing plasmid, IncP-1, Tra, RepA(Ts); Ap^r^ | Voisard et al. 1988 |
| pME3087 | Suicide vector, ColE1 replicon, Mob; Tc^r^ | Voisard et al. 1994 |
| pME3087phlD | pME3087 containing a *Bam*HI/*Hin*dIII *phlD* region with a deletion of 720 bp in the *phlD* gene; Tc^r^ | This study |
| pME3087-1348 | pME3087 containing a *Bam*HI/*Hin*dIII *1348* region with a deletion of 228 bp in the *1348* gene; Tc^r^ | This study |
| pME3087-1349 | pME3087 containing a *Bam*HI/*Hin*dIII *1349* region with a deletion of 408 bp in the *1349* gene; Tc^r^ | This study |
| pME3087-1350 | pME3087 containing a *Bam*HI/*Hin*dIII *1350* region with a deletion of 897 bp in the *1350* gene; Tc^r^ | This study |
| pME3087-1351 | pME3087 containing a *Bam*HI/*Hin*dIII *1351* region with a deletion of 1101 bp in the *1351* gene; Tc^r^ | This study |
| pME3087-1353 | pME3087 containing a *Bam*HI/*Hin*dIII *1353* region with a deletion of 2316 bp in the *1353* gene; Tc^r^ | This study |
| pME3087c1348 | pME3087 containing a 1348 gene; Tc^r^ | This study |
| pME6014 | Cloning vector for the construction of translational *'lacZ* fusion; Tc^r^ | Schnider-Keel et al. 2000 |
| pME6014-1348 | Translational *1348'-'lacZ* fusion under p*tac*; Tc^r^ | This study |
| pME6031 | pACYC177-pVS1 shuttle vector; Tc^r^ | Heeb et al. 2000 |
| pME6031-OS3 | pME6031 containing *1348-1351* genes; Tc^r^ | This study |

REFERENCES

Heeb, S., Itoh, Y., Nishijyo, T., Schnider, U., Keel, C., Wade, J., et al. (2000) Small, stable shuttle vectors based on the minimal pVS1 replicon for use in gram-negative, plant-associated bacteria. *Mol. Plant Microbe Interact.* 13,232-237. doi: 10.1094/MPMI.2000.13.2.232

Morohoshi, T., Yamaguchi, T., Xie, X., Wang, WZ., Takeuchi, K., and Someya, N. (2017) Complete genome sequence of *Pseudomonas chlororaphis* subsp. aurantiaca reveals a triplicate quorum-sensing mechanism for regulation of phenazine production. *Microbes Environ*. 32,47-53. doi: 10.1264/jsme2.ME16162

Sambrook, J., Russell, D.W., and Irwin, N. (2001) Molecular Cloning: A Laboratory Manual, 3rd ed. New York: Cold Spring Harbor Laboratory Press.

Voisard, C., Rella, M., and Haas, D. (1988) Conjugative transfer of plasmid RP1 to soil isolates of *Pseudomonas fluorescens* is facilitated by certain large RP1 deletions. *FEMS Microbiol. Lett.* 55,9-13. doi: 10.1111/j.1574-6968.1988.tb02790.x

Voisard, C., Bull, C.T., Keel, C., Laville, J., Maurhofer, M., Schnider, U., et al., (1994) Biocontrol of root diseases by *Pseudomonas fluorescens* CHA0: current concepts and experimental approaches. In: Molecular Ecology of Rhizosphere Microorganisms (O'Gara, F., Dowling, D.N., and Boesten, B., eds): VCH Weinheim, Germany. pp. 67-89. doi: 10.1002/9783527615810.ch6

**Supplementary Table S2**

Oligonucleotides used in the present study

| Oligonucleotide | Description | Source or o reference |
| --- | --- | --- |
| 1348UF | 5'-ACGTGGATCCGGTTGATGTAGAAGTTGCG-3',  underlining indicates the artificial *Bam*HI site | This study |
| 1348UR | 5'-CGTACTCATGGTATCTCTCC-3',  anneals to the 5' region of 1348DF | This study |
| 1348DF | 5’-GGAGAGATACCATGAGTACGCAACAGGCGCGAATGGACTGA-3' | This study |
| 1348DR | 5'-ACGTAAGCTTTGACAGTTCCGCATATAACG-3',  underlining indicates the artificial *Hin*dIII site | This study |
| 1349UF | 5'-ACGTGGTACCTTTGGGCGATTAACGAACAC-3',  underlining indicates the artificial *Kpn*I site | This study |
| 1349UR | 5'-GAAACGCTCGAAGTCGGCTT-3',  anneals to the 5' region of 1349DF | This study |
| 1349DF | 5’-AAGCCGACTTCGAGCGTTTCAAGCCCTTGCCGTGAACTTT-3' | This study |
| 1349DR | 5'-ACGTAAGCTTCCAGATCGAGTATCGAGTT-3',  underlining indicates the artificial *Hin*dIII site | This study |
| 1350UF | 5'-ACGTGGATCCAACAGGCGCGAATGGACTGA-3',  underlining indicates the artificial *Bam*HI site | This study |
| 1350UR | 5'-AGTTCCGCATATAACGCGAC-3',  anneals to the 5' region of 1350DF | This study |
| 1350DF | 5’-GTCGCGTTATATGCGGAACTAATCTGCTGCAGGAGGTGCAT-3' | This study |
| 1350DR | 5'-ACGTAAGCTTAGGTACTCGCGCAAGTCGTA-3',  underlining indicates the artificial *Hin*dIII site | This study |
| 1351UF | 5'-ACGTGGATCCTGAATCTGACCTTCCCTGAG-3',  underlining indicates the artificial *Bam*HI site | This study |
| 1351UR | 5'-CTTTTCCACTTGCAGCAGATCG-3',  anneals to the 5' region of 1351DF | This study |
| 1351DF | 5’-CGATCTGCTGCAAGTGGAAAAGTTGGCATGGACTCAAGTGG-3' | This study |
| 1351DR | 5'-ACGTAAGCTTGAAACCAAGATGGG-3',  underlining indicates the artificial *Hin*dIII site | This study |
| 1353UF | 5'-ACGTGGATCCTGCAGAAAGTCGGCGACAA-3',  underlining the indicates artificial *Bam*HI site | This study |
| 1353UR | 5'-GTTACTTTCCGAGGACACGT-3',  anneals to the 5' region of 1353DF | This study |
| 1353DF | 5’-ACGTGTCCTCGGAAAGTAACTTGTCCCTGCTGATGGTGTT-3' | This study |
| 1353DR | 5'-ACGTAAGCTTGAGGTCGCGGTTGTTTT-3',  underlining indicates the artificial *Hin*dIII site | This study |
| phlDUF | 5'-ACGTGGATCCGACAACCAGGTCAAGG-3',  underlining indicates artificial *Bam*HI site | This study |
| phlDUR | 5'-TTGCGGAAACATGACGTGTG-3',  anneals to the 5' region of phlDDF | This study |
| phlDDF | 5’-CACACGTCATGTTTCCGCAACTCAACTACGACAGCTTCGA-3' | This study |
| phlDDR | 5'-ACGTAAGCTTGGTGACAATGATGCTGGT-3',  underlining indicates the artificial *Hin*dIII site | This study |
| 1348proF | 5'-ACGTGAATTCTTTGGGCGATTAACGAACAC-3',  underlining indicates the artificial *Eco*RI site | This study |
| 1348proR | 5'-ACGTGGATCCTCGTACTCATGGTATCTCTC-3',  underlining indicates the artificial *Bam*HI site | This study |
| 3087-1348U | 5'-AGTCGACCTGCAGGCATGCAATGTAGAAGTTGCGCAGGTTG -3',  underlining anneals to the 3' region of pME3087 cut with *Hin*dIII | This study |
| 3087-1348D | 5'-AATCCGGTGAGAATGGCAAAAGTTCCGCATATAACGCGACAAAC -3',  underlining anneals to the 5' region of pME3087 cut with *Hin*dIII | This study |
| OS3F | 5'-ACGTAAGCTTTGGGCGATTAACGAACAC-3',  underlining indicates the artificial *Hin*dIII site | This study |
| OS3R | 5'-ACGTGGTACCTGTTCTGAAACAGGATCC-3',  underlining indicates the artificial *Kpn*I site | This study |
| 25F1348 | 5'-GAAGGAGATATACATATGAGTACGAAAGTCAATCAGGAG-3' | This study |
| 25R1348 | 5'-CTCGAATTCGGATCCTCAGTCCATTCGCGCCTGTTGCTG-3' | This study |
